# Human Pluripotent Stem Cell-Derived Micropatterned Ectoderm Allows Cell Sorting of Meso-Endoderm Lineages

**DOI:** 10.3389/fbioe.2022.907159

**Published:** 2022-07-22

**Authors:** Yang Yang, Cecilia Laterza, Hannah T. Stuart, Federica Michielin, Onelia Gagliano, Anna Urciuolo, Nicola Elvassore

**Affiliations:** ^1^ Department of Industrial Engineering (DII), University of Padova, Padova, Italy; ^2^ Fondazione Ricerca Biomedica Avanzata Onlus, Veneto Institute of Molecular Medicine, Padova, Italy; ^3^ Great Ormond Street Institute of Child Health, University College London, London, UK; ^4^ Department of Molecular Medicine, University of Padova, Padova, Italy; ^5^ Istituto di Ricerca Pediatrica, Città della Speranza, Padova, Italy

**Keywords:** micropatterning, cell sorting, ectoderm, hPSC differentiation, human *in vitro* model

## Abstract

The human developmental processes during the early post-implantation stage instruct the specification and organization of the lineage progenitors into a body plan. These processes, which include patterning, cell sorting, and establishment of the three germ layers, have been classically studied in non-human model organisms and only recently, through micropatterning technology, in a human-specific context. Micropatterning technology has unveiled mechanisms during patterning and germ layer specification; however, cell sorting and their segregation in specific germ layer combinations have not been investigated yet in a human-specific *in vitro* system. Here, we developed an *in vitro* model of human ectodermal patterning, in which human pluripotent stem cells (hPSCs) self-organize to form a radially regionalized neural and non-central nervous system (CNS) ectoderm. We showed that by using micropatterning technology and by modulating BMP and WNT signals, we can regulate the appearance and spatial distribution of the different ectodermal populations. This pre-patterned ectoderm can be used to investigate the cell sorting behavior of hPSC-derived meso-endoderm cells, with an endoderm that segregates from the neural ectoderm. Thus, the combination of micro-technology with germ layer cross-mixing enables the study of cell sorting of different germ layers in a human context.

## Introduction

The process of gastrulation is the basis of the generation of all organs and is one of the most critical steps during development (Krens and Heisenberg, 2011). During gastrulation, the body plan is created and organ primordial form. In humans, this commences in the week immediately after the implantation of the embryo on the uterus and is thus inaccessible to study. At the cellular level, identities change and diversify as differentiation occurs into the three germ layers, while at the tissue level, appropriate cell sorting and spatial patterning must occur within the first 3 weeks after conception (pcw). These early post-implantation processes contribute to the formation of different organs (Krens and Heisenberg, 2011). These phenomena have been classically studied in model organisms, but mechanisms do not always reproduce in human settings, and human development is much more prone to failure at this sensitive stage than the widely used mouse model (Wamaitha and Niakan, 2018).

Thanks to the development of *in vitro* culture of human pluripotent stem cells (hPSCs), which can differentiate into the three germ layers, and the use of microtechnologies, researchers have begun to create *in vitro* structures which can capture some features of early human embryo development (Srivastava and Kilian, 2019; Baillie-benson et al., 2020). Micropatterning technology, which enables geometric confinement of hPSCs *in vitro,* has emerged as a method of choice for studying signaling dynamics and cell-fate patterning in early human gastrulation (Warmflash et al., 2014), reproducing *in vitro* the critical stages of gastrulation occurring within the first three pcw of development (Warmflash et al., 2014; Baillie-benson et al., 2020; Minn et al., 2020). Although these 2D micropatterns do not faithfully recapitulate 3D morphogenesis in all its complexity, they can be useful in capturing specific phenomena which require a 2D setting. For instance, micropatterns can allow dissecting the role of spatial and geometric constraints in cell fate decisions, which could not be unveiled in standard adhesive conditions or 3D suspension culture (Warmflash et al., 2014; Baillie-benson et al., 2020).

Several micropatterning techniques have been developed for studies in which the cell colony geometry needs to be controlled. Among these, the photo-patterning method allows the *ad hoc* formation of cell adhesive regions with defined shape and size on a glass surface by the spatially controlled exposure of photosensitive materials to UV light (between 200–385 nm) through a photomask with defined micro-features (Théry and Piel, 2009).

Geometrically confined hPSC colonies treated with appropriately chosen combinations of morphogens generate radially symmetric patterns of different cell types, recapitulating some aspects of the patterning of germ layers at gastrulation or ectodermal cell fates at neurulation stages (Peerani et al., 2007; Warmflash et al., 2014; Deglincerti et al., 2016; Etoc et al., 2016; Tewary et al., 2017; Britton et al., 2019; Chhabra et al., 2019; Heemskerk et al., 2019; Martyn et al., 2019; Karzbrun et al., 2021). In addition, when these morphogens are provided to geometrically confined hPSCs using gradients generated via a microfluidic approach, the intrinsic radial symmetry is broken and differentiated cells arrange into distinct domains (Manfrin et al., 2019).

Another morphogenic process occurring during early embryonic development, which is crucial for the proper segregation of different cell layers, involves the so-called “cell sorting” mechanism. Through cell sorting, the different germ layers achieve segregation to ensure the formation of tissue boundaries (Fagotto, 2014). This phenomenon has been widely demonstrated in model organisms but was only recently shown for the first time in human cells by micropatterning technology (Minn et al., 2020). In that work, geometrically confined hPSCs were induced to differentiate into radially organized germ layers *via* BMP4 stimulation (Warmflash et al., 2014; Minn et al., 2020). Upon gastruloid dissociation and re-seeding onto micropatterns, cells belonging to different germ layers segregated from each other, creating islands of layer-specific aggregates, confirming that human gastruloids exhibit evolutionarily conserved sorting behavior (Minn et al., 2020). In particular, ectodermal cells are segregated from endodermal cells, but mixed with mesodermal cells during human development (Minn et al., 2020). However, in this study, the authors did not investigate the ability of human-derived cells to spatially segregate within pre-patterned structures, nor analyzed the different behavior of single lineages within the ectoderm germ layer.

Here, we adapted the 2D micropatterned ectoderm culture and validated the cell sorting ability of meso-endoderm lineages when co-seeded onto a radial pre-patterned ectoderm derived from hPSCs.

We first performed ectoderm patterning since it is an epithelial tissue and thus needs to form its correct tissue packing and apical/basal/lateral geometry as a community. By using photo-micropatterning technology, we showed the ability of geometrical confinement to induce the formation of a radially regionalized neural and non-central nervous system (CNS) ectoderm, and that the spatial distribution of these regions was dependent on the signals provided in the culture. Afterward, we seeded mesendoderm cells on top since they are of non-epithelial, motile mesenchymal lineage and showed that the pre-patterned ectoderm can guide the cell sorting behavior of hPSC-derived meso-endoderm cells. Specifically, the endoderm tends to segregate from the neural ectoderm and preferentially localizes in non-CNS ectoderm regions, whereas the mesoderm shows a more spread localization within micropatterns. By performing in this order, we demonstrate the possibility of using this micropatterning tool as a model to study dynamic organizational processes such as tissue sorting, differential adhesion, and cell migration on top of a defined base of distinct identity domains.

Thus, this study shows that by modulating the signaling molecules provided to geometrically confined hPSCs, we can perturb the ectoderm micropattern formation and that the micropattern system can be used to study the interaction and cell sorting properties of different germ layers.

## Results

### Photo-Patterning Method Allows the Generation of Reproducible hPSC Micropatterns

**FIGURE 1:**
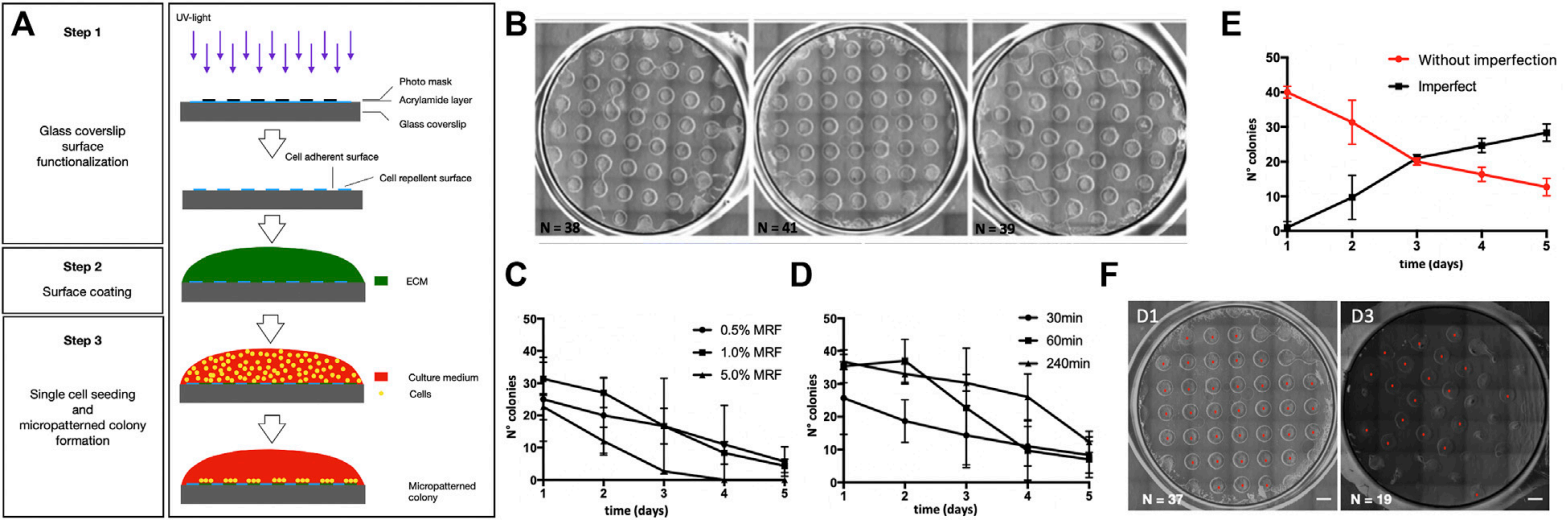
Micropatterning technology for human iPSC geometric confinement. **(A)** Schematic representation of the micropatterning strategy which includes step 1: glass coverslip surface functionalization; step 2: Poly-L-lysine and Matrigel surface coating; and step 3: single-cell seeding and micropatterned colony formation. **(B)** Brightfield images of three independent batches showing a reproducible yield in the number of well-formed micropatterns per coverslip (12 mm diameter) using human pluripotent stem cells (iPSCs). **(C,D)** Optimization of the Matrigel concentration and time of rock inhibitor withdrawal. Graph showing quantification of the number of well-organized micropatterned iPSC colonies during 5 days modifying Matrigel coating concentration **(C)** and the time of Rock inhibitor withdrawal **(D)**. **(E)** Number of colonies with intact round geometry (red, named without imperfection) and with non-preserved geometrical integrity (black, named imperfect) during 5 days of differentiation by applying the established method (1% Matrigel coating, Rock inhibitor withdrawal at 240 min). Data derived from at least three independent experiments (three coverslips per experiment). Error bars represent the standard error. **(F)** Representative images of micropatterns after 1 (D1) or 3 (D3) days from seeding. Scale bar = 1 mm.

We developed a micropatterning method used for human pluripotent stem cell (hPSC) culture and ectoderm induction (Figure 1A). Briefly, the micropatterning method allows the generation of cell-adhesive areas of defined size and shape via the use of photomasks (Figure 1A). First, we confirmed that we were able to generate hPSC colonies of 1 mm diameter on a glass coverslip (12 mm diameter) glass coverslip (Figure 1B) corresponding to the photo-preserved areas.

To find the best experimental conditions to generate micropatterns, we kept the size and shape of the micropatterns constant (round shapes of 1 mm diameter) and tested two different influencing factors: *1*) Matrigel growth factor reduced (MRF) coating concentration to optimize cell adhesion, and *2*) the time of ROCK (Rho-associated protein kinase) inhibitor (Ri) exposure to enhance cell viability after seeding. We tested three different concentrations of MRF (0.5, 1.0, and 5.0% v/v) (Figure 1C) and three different times of Ri withdrawal after seeding (30, 60, and 240 min) (Figure 1D) and counted the number of well-organized colonies over time (i.e., a colony of 1 mm diameter with intact borders). From the results of these tests, we selected an MRF concentration of 1% and Ri treatment timing of 240 min. Next, we evaluated the stability of the micropatterns over time, since the integrity of the micropattern’s geometry is a requirement to increase the reproducibility of cell patterning. We longitudinally analyzed the appearance of the colonies and found that we were able to maintain at least 10 colonies showing an intact round geometry for up to 5 days (Figure 1E).

### Combination of Different Signaling Molecules Influences the Spatial Distribution of Geometrically Confined hPSCs Patterned Toward Ectoderm Lineages

During development, the ectoderm is patterned by a combination of BMP and WNT signaling. We hypothesized that upon specific and time-regulated stimulation with BMP, TGFβ, and WNT inhibitors coupled with the controlled administration of exogenous BMP, the micropatterned hPSCs could self-organize into different cells fates within the ectoderm cell populations. First, we confirmed the ability of dual SMAD inhibition in promoting the pluripotency exit and acquisition of homogeneous neuroectoderm fate in standard culture conditions (Supplementary Figure S1A). We found that the neural ectodermal markers PAX6 and SOX1 started to be expressed significantly from day 3 and reached the peak between days 4 and 5, while the pluripotency marker OCT4 was highly expressed on day 1, decreased during the following days, and completely disappeared between days 4 and 5 of the culture (Supplementary Figures S1B,C). SOX2, instead, was stably expressed during the whole 5 days since it is a shared marker between the pluripotency and neural ectoderm (Feng and Wen, 2015) (Supplementary Figures S1B,C).

**FIGURE 2:**
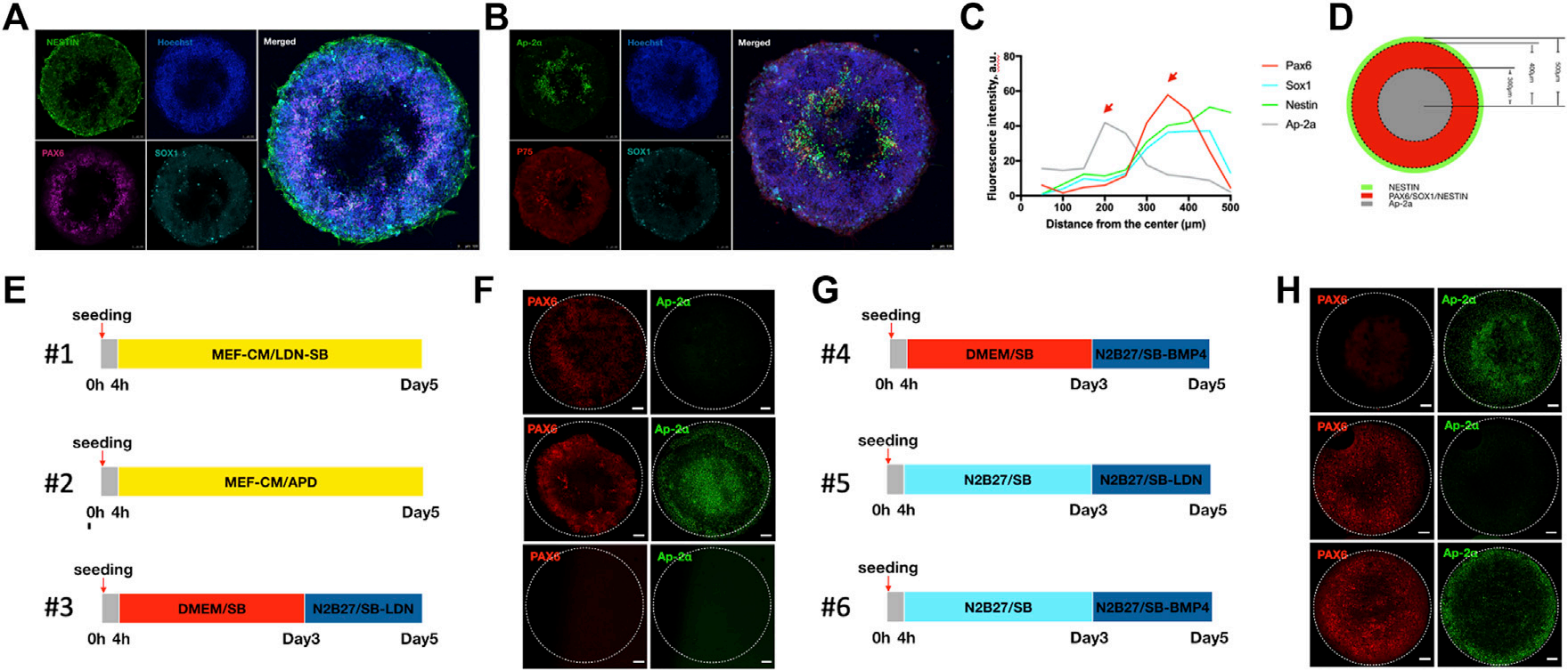
Effect of BMP and WNT signaling on neural ectoderm patterning. **(A–D)** Representative images of micropatterns obtained inducing neural differentiation *via* exposure with APD mix (A83-01, PNU74654, and Dorsomorphin) in MEF-conditioned medium (MEF-CM) (protocol n2). Shown are immunostaining for the neural ectoderm NESTIN (green), PAX6 (purple), and SOX1 (light blue) **(A)**, and neural crest markers AP2a (green) and p75 (red) **(B)** and nuclei are counterstained with Hoechst (blue). **(C)** Graph showing the spatial distribution of neural ectoderm markers (PAX6, SOX1, and NESTIN) and neural crest markers (AP2a) under geometric confinement expressed as average nuclear intensities of the indicated marker as a function of distance from the colony center. **(D)** Schematic representation of the distribution of neural ectodermal and neural crest populations within micropatterns. **(E–H)** Schematic representation of six different combinations of signaling molecules (TGFb inhibitors; BMP inhibitors; and WNT inhibitors) and basal media within 5 days of the experiment. **(E,G)** Representative images of neural patterned human iPSCs by applying different induction protocols on day 5 of culture. **(F,H)** Shown are Pax6 (red, neuroectoderm marker) and AP-2α (green, neural crest marker). Scale bar = 100 μm. The dashed white circles indicate the region of micropatterns. N> = 5. MEF-CM = MEF-conditioned media; LDN = LDN193189; SB = SB431542; APD = A83-01, PNU74654, and Dorsomorphin; Ri = Rock inhibitor.

Then we applied TGFβ, WNT, and BMP inhibition to micropatterned hPSCs using a combination of small molecules named hereafter APD (i.e., Α83-01, PNU74654, and Dorsomorphin). As shown in Figures 2A–D, upon ectoderm induction on micropatterns of 1 mm diameter, we obtained radial segregation of the neural ectoderm in the outer region of the pattern (Figure 2A) with a peak of fluorescence intensity associated with PAX6 at 370 μm from the center (Figure 2C). In addition, we observed the appearance of a non-CNS ectoderm population (defined by AP2α positivity) (Tchieu et al., 2017) in the center of the pattern (Figure 2B) with a peak fluorescence intensity associated with AP2α at 200 μm from the center (Figure 2C). Thus, instead of a homogeneous layer of neural ectoderm as observed in standard culture conditions (Supplementary Figures S2B,C), the geometric confinement resulted in the generation and segregation of two different cell populations: a neural ectoderm in the outer region and a non-CNS ectoderm in the inner part of the pattern (Figure 2D).

Then we tested whether different combinations of WNT and BMP signals provided to geometrically confined hPSC colonies in a time-regulated fashion affect the appearance and spatial segregation of multiple ectodermal fates (Figures 2E–H). We tested six different conditions based on previously published protocols for human ectoderm patterning (Tchieu et al., 2017; Xue et al., 2018; Britton et al., 2019). As expected, changing the stimuli had a substantial impact on the micropattern formation and multiple ectoderm fates’ acquisition: continuous TGFβ and BMP inhibition (#1) led to the formation of an external ring of the neural ectoderm (PAX6 positive cells); continuous TGFβ, BMP, and WNT inhibition (#2) led to the segregation of two distinct populations (neural ectoderm, PAX6 positive, and non-neural ectoderm AP2α positive) with a central core of the non-neural ectoderm and an outer ring of the neural ectoderm; two-step induction with continuous TGFβ inhibition and inhibition of BMP from day 3 in DMEM-based medium (#3) abolished the formation of both populations, whereas the same combination of small molecules in an N2/B27-based medium (#5) led to the formation of a homogeneous neural ectoderm pattern; two-step induction with continuous TGFβ inhibition and the addition of BMP4 from day 3 in the DMEM-based medium (#4) led to the formation of a central core of the non-neural ectoderm within the micropatterns, whereas the same combination of small molecules in the N2/B27-based medium (#6) led to the segregation of the two distinct ectoderm populations but with inverted localization compared to condition #2, with the vast majority of the pattern covered by the neural ectoderm and a thin outer ring of the non-neural ectoderm (Figures 2F,H).

Thus, not only the temporally controlled perturbation of BMP and WNT signaling via specific inhibitors but also the stimuli contained within the basal media, among which BMP (secreted by MEFs or present in the KSR of DMEM) (Xu et al., 2005) could play a major role in defining the presence and spatial distribution of different fates within the ectoderm lineage.

### Selective Cell Sorting Behavior of Meso-Endodermal Cells Reseeded Onto the Ectoderm Micropatterns

Then we investigated whether this micropatterning technology would be a suitable platform to create multilayer systems and to investigate the interaction between cells belonging to different germinal layers in terms of selective cell sorting (Minn et al., 2020). In particular, we tested whether the hPSC-derived meso-endoderm can undergo cell sorting *in vitro* on pre-existing patterned ectoderm structures as previously observed for human gastruloid-derived cells (Minn et al., 2020). To distinguish the two cell populations, the meso-endoderm was derived from a GFP-expressing hPSC line.

We used a published protocol to generate a homogeneous meso-endoderm layer of cells in 48 h (Supplementary Figure S1A) (Giobbe et al., 2015) and confirmed the prevalence of the mesoderm marker T/Brachyury at 24 h of differentiation and the endoderm marker SOX17 at 48 h of differentiation (Supplementary Figures S1B,C).

Based on these data, we harvested meso-endoderm cells at 24 h of differentiation and plated them onto 5-day-old micropatterned ectoderm structures. We tested three of the protocols described in Figure 2F, which gave the most extreme results (i.e., #2 with an outer ring of PAX6 and an inner core of AP2α; #4 with only an inner core of AP2α; and #6 an inner core of PAX6 and an outer ring of AP2α) and co-cultured with meso-endodermal cells for an additional 3 days (Figure 1A).

**FIGURE 3:**
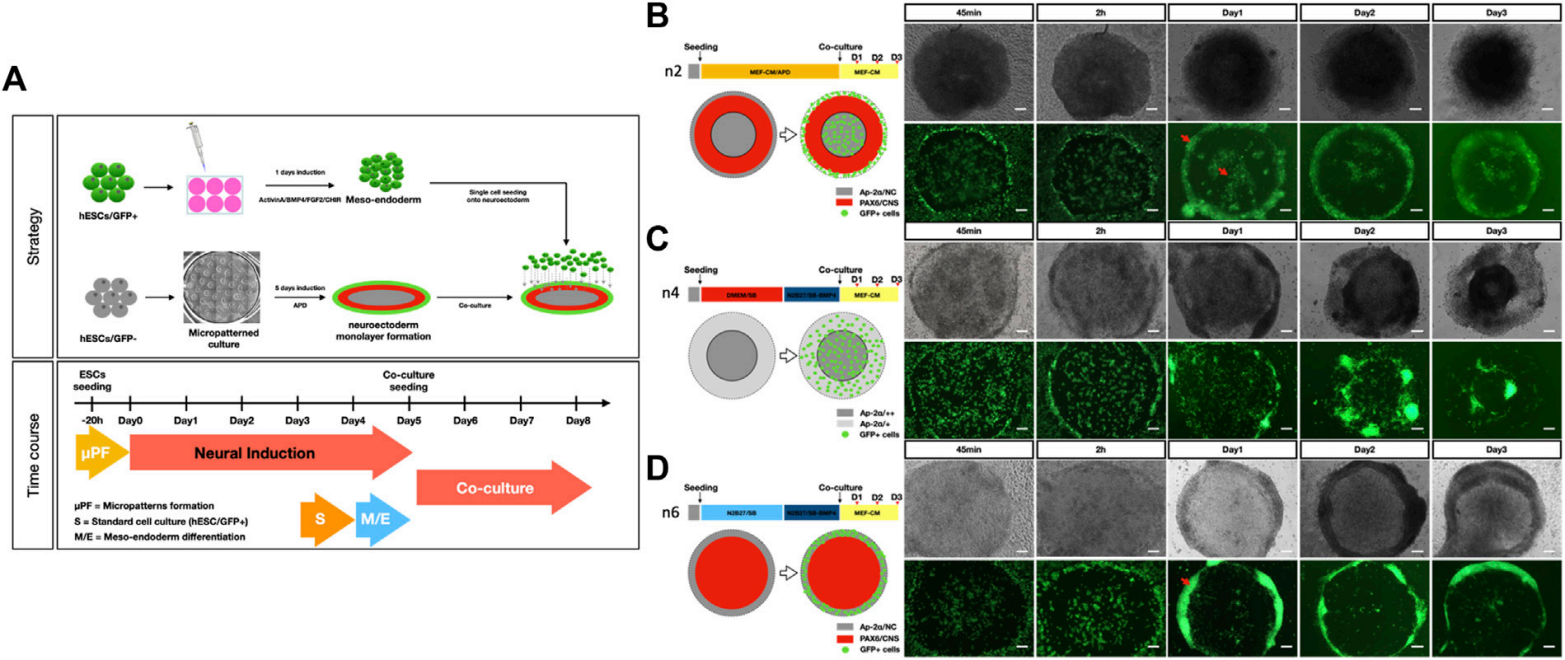
Spatial distribution of the meso-endoderm on different neuroectoderm micropatterns. **(A)** Schematic representation of the co-culture strategy of human iPSC micropatterned toward the neural ectoderm and human iPSC-derived GFP + meso-endoderm M-E. **(B–D)** Interaction between three extreme conditions of neuroectoderm micropatterning and GFP + meso-endoderm. Schematic representation of the different patterning conditions and time course from seeding to day 3 of co-culture. **(B)** In the n2 condition, which gives rise to the central NC population (AP2a-positive), the M-E is localized within and outside the PAX6-positive ring; **(C)** in the n4 condition, in which there is no PAX6-positive population at day 5 and the pattern is made of AP2a-positive cells, M-E spread on top of the patterns; **(D)** in n6 condition, in which there is only a PAX6-positive population and no AP2a, M-E localizes at the edge of the patterns. Pax6 (CNS marker) and AP-2α (NC marker, “+ +” represents relatively high expression and “+” represents relatively low expression). Scale bar = 100 μm N > = 5.

To monitor the distribution of GFP-positive meso-endoderm cells under the three different micropattern conditions, we followed the co-cultures over time (Figures 3B–D). Immediately after seeding (45 min and 2 h), GFP-positive cells were evenly distributed in all three conditions (Figures 3B–D). Already at 1 day after seeding, we observed major differences which were maintained until day 3. In condition #2 (Figure 1B), the GFP-positive meso-endoderm was localized in the middle and at the outer border of the pattern (Figure 3B). In condition #4 (Figure 3C), the GFP-positive meso-endoderm spread all over the pattern. In condition #6 (Figure 3D), the GFP-positive meso-endoderm created an outer ring. Although the spatial distribution of the meso-endoderm differed among the three conditions, they all shared the common tendency of meso-endoderm cells to avoid regions occupied by the neural ectoderm (PAX6-positive) and accumulate in regions occupied by the non-CNS ectoderm (AP2a) (Figures 1B–D and Figures 3B–D).

During *in vivo* development, the ectoderm and endoderm are spatially separated by the presence of the mesoderm (Solnica-Krezel and Sepich, 2012). Human gastruloids dissociated and reseeded on micropatterns showed the ability to self-organize in segregated cell clusters with ectodermal cells more associated with the mesoderm and completely segregated from the endoderm, probably through selective cadherin expression (Minn et al., 2020). Based on this evidence, we tested whether the mesoderm (T/Brachyury+) and the endoderm (SOX17+) populations show a differential distribution on ectoderm patterns (condition n2) after 3 days of co-culture.

**FIGURE 4:**
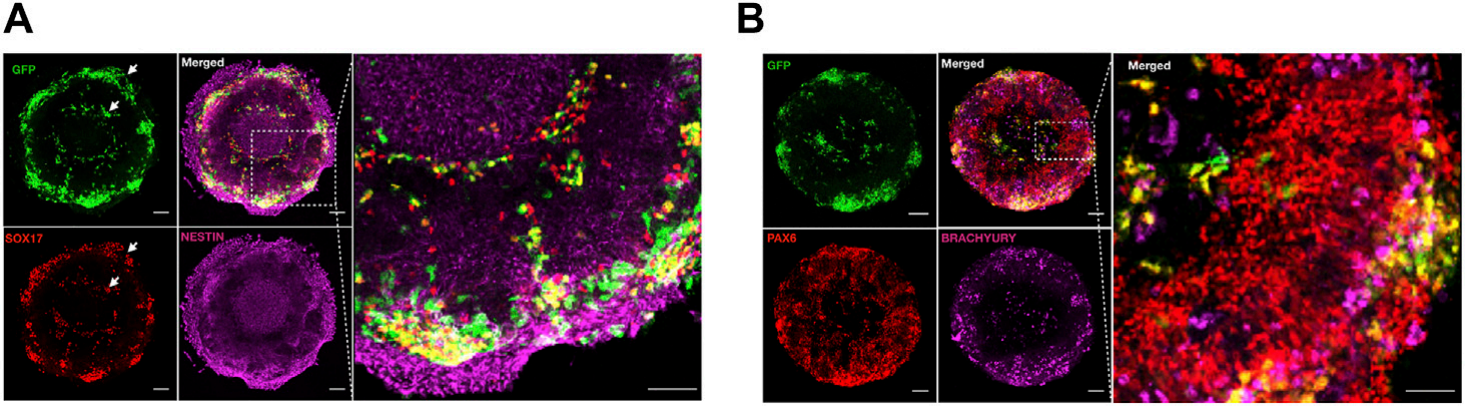
Cell sorting of meso-endoderm cells on micropatterned ectoderm culture. Representative images of meso-endoderm at day 3 after seeding on ectoderm micropatterns stained for GFP (green) to visualize the meso-endoderm, SOX17 (red) to the mark endoderm, and Nestin (purple) to mark the neural ectoderm **(A)**, or coupled with PAX6 (red) to mark the neural ectoderm and Brachyury (purple) to mark the mesoderm **(B).** Scale bar = 100 μm.

We found that both GFP + SOX17 + endoderm and GFP + T/Brachyury + cells preferentially clustered at the edges of the areas of micropatterns occupied by the non-CNS ectoderm (Figure 4A), and GFP + T/Brachyury + cells partially overlapped with regions covered by the neural ectoderm as previously observed (Fagotto, 2014) (Figure 4B).

## Discussion

In this study, we developed a micropatterning method in which hPSCs can self-organize to create radially distributed patterns of neural and non-neural ectoderm. As recently published (Xue et al., 2018; Britton et al., 2019), the formation of different territories within geometrically confined hiPSC colonies could be mainly attributed to the generation of a gradient of endogenous BMP signaling, which is activated by mechanical stimuli at the edge of the colonies and then gradually spread toward the center of the pattern (Xue et al., 2018) and plays a pivotal role in providing positional information in the ectoderm during neurulation (Tchieu et al., 2017). Indeed, high levels of BMP differentiate the ectoderm into the epidermis, low levels of BMP differentiate the ectoderm into the neural plate and intermediate BMP levels give rise to neural plate border specification (Tchieu et al., 2017). The mechanism that links the BMP dose to ectodermal lineage specification seems to be related, at least in part, to the direct effect of BMP on the upregulation of AP2a, which in turn can work together with WNT and FGF pathways to specify the different regions of the non-neural ectoderm (Tchieu et al., 2017). In our experimental setting, we obtained consistent segregation between the neural ectoderm PAX6-positive in the outer border of the pattern and the non-CNS ectoderm AP2α-positive in the central region, which is in contrast with previous publications where the non-CNS ectoderm localized at the colony border and the neural ectoderm at the center (Xue et al., 2018). This major difference in the spatial distribution could be in part attributed to the cell density and the size of the patterns. Indeed, in the context of ectoderm specification, only increasing the colony size from 500 to 800 μm has been reported to be sufficient to revert the localization of the neural and non-CNS ectoderm, with patterns of 800 μm showing a spatial distribution comparable to our 1,000 μm patterns (Xue et al., 2018).

Given the high proliferation state of the neural ectoderm, the high rate of cell death observed during neural ectoderm commitment and the effect of cell density on the specification of the CNS and the non-CNS ectoderm, future time-lapse experiments could help in following better the dynamics of spatial patterning shaping.

Not only endogenous but also exogenous signals play a central role in guiding symmetry breaking and lineage specification. So, we modified the neural induction medium to investigate the role of WNT and BMP pathways in guiding ectoderm lineage specification and spatial distribution within the micropatterns. First, keeping the same basal media (KSR-based medium conditioned by MEF), we only varied the inhibitors used for the whole 5 days of differentiation: when we inhibited BMP and TGFβ-mediated SMAD activation, we only obtained neural ectoderm specification but not non-CNS ectoderm induction; whereas, also adding an inhibitor of WNT signaling, we obtained segregation between the neuroectoderm in the outer region of the pattern and the non-CNS ectoderm in the central area. The addition of a WNT inhibitor to a dual SMAD inhibitory cocktail should enhance neural ectoderm specification. However, since we are using a KSR-based medium conditioned by MEF, which is known to contain BMP, we can speculate that the presence of BMP in addition to WNT inhibition could have induced placode specification, which homogeneously expresses the AP2α marker within the non-CNS ectoderm territory (Tchieu et al., 2017). Indeed, the presence of AP2α-expressing cells within the center of the micropatterned hPSCs has already been reported upon WNT inhibitor treatment, and the width and localization of an AP2α-expressing territory were directly correlated with the level of BMP (Britton et al., 2019).

It has been previously demonstrated that providing BMP4 to geometrically confined hPSCs with proper timing allows the generation of multiple ectoderm fates (Britton et al., 2019). So, to shed light on the role of the BMP pathway on neural and non-neural ectoderm patterning, we tested four combinations of the two-step induction protocols: after 3 days of ectoderm induction in the presence of a TGFβ inhibitor only, hPSC patterns have been either supplemented with BMP4 or depleted of BMP via LDN treatment (a selective BMP inhibitor). We also tested the influence of the factors contained within the basal medium used and we performed this experiment in the DMEM 20% KSR medium (named DMEM), known to have a BMP activity-inducing property, (Xu et al., 2005) or in the N2/B27-based medium, which is known to be devoid of BMP.

The inhibition of BMP vial LDN treatment in the context of a two-step induction protocol using a KSR-based medium completely abolished the formation of ectoderm patterning and the appearance of the AP2α-expressing territory. However, probably because of the short time of the induction (48 h) in combination with the patterns’ size, the PAX6 neural ectoderm did not appear. Indeed, in our standard culture conditions, we need at least 5 days to obtain high levels of PAX6 expression upon LDN treatment. On the other hand, by replacing LDN with BMP4 treatment, the AP2α-expressing territory spread in all the micropatterns, and neural ectoderm differentiation was completely inhibited, in accordance with published data (Xue et al., 2018).

In contrast, replacing the KSR medium with the N2/B27 medium, a condition known to be devoid of BMP signaling, and providing LDN, was sufficient to promote neural ectoderm differentiation across the entire colony at the expense of non-CNS AP2α-positive cells. By replacing LDN with BMP4, we restored the ectoderm patterning with a core of the neural ectoderm and an outer region of the non-CNS ectoderm as previously published (Deglincerti et al., 2016; Etoc et al., 2016; Xue et al., 2018; Britton et al., 2019).

Finally, we leveraged our *in vitro* system to dissect the selective cell-sorting behavior of human meso-endoderm cells once seeded onto the pre-patterned ectoderm.

Cell sorting has been widely described *in vivo* and *in vitro* in different model systems as a crucial mechanism to ensure germ layer boundary formation (Krens and Heisenberg, 2011) and has also been described in human blastocyst-like structures and gastruloids *in vitro* (Shahbazi and Zernicka-Goetz, 2018; Minn et al., 2020). However, the ability of single or multiple human germ layers to self-organize onto already pre-patterned structures has never been investigated before.

We took advantage of three of the ectoderm patterns that showed a distinct distribution of the neural and non-neural ectoderm and we observed a conserved tendency of the meso-endoderm to preferentially distribute onto the non-neural ectoderm rather than the neural ectoderm. When we investigated in detail, we found that the endoderm tended to completely segregate from the neural ectoderm, preferentially occupying regions of the non-CNS ectoderm, whereas mesoderm distribution was spread more onto the patterns, and also in regions occupied by the neural ectoderm (Fagotto, 2014). These data have never been reported so far. Indeed, it has been already reported that the ectoderm and endoderm tend to segregate (Minn et al., 2020), but the authors have not distinguished between the neural ectoderm and non-neural ectoderm. Since the non-neural ectoderm is known to undergo epithelial to mesenchymal transition and to share features with the mesoderm, like the expression of defined adhesion molecules (among which cadherins play a central role) (Taneyhill, 2008; Manohar et al., 2020), we hypothesize that when we seeded meso-endoderm cells onto our patterning system, these cells tended to segregate onto mesodermal-like cells probably because of their differential expression in cadherins. Cell sorting behavior can be explained via three distinct models: differential adhesion, differential cortical tension, and contact inhibition (Fagotto, 2014); however, differential adhesion has been identified as the prevailing mechanism for human gastruloids (Minn et al., 2020). In this system, the authors found that SOX17 + endoderm expresses higher levels of classical cadherins that promote cell adhesion via homophilic and heterophilic interactions (CDH1, CDH2, and CDH3), protocadherin PCDH1, and EPCAM (Takeichi, 1990), while the ectoderm, mesoderm, and extra-embryonic clusters expressed higher levels of CDH11 (Minn et al., 2020). Interestingly, CDH11 is highly expressed in non-neural ectoderm and controls multiple essential cellular functions in neural crest cells (Manohar et al., 2020) confirming also in our system the differential adhesion as a possible mechanism underlying the cell sorting behavior.

Our work provides proof-of-principle that the sophisticated control of lineage location with micropatterns allows us to study the simultaneous interaction between multiple human lineages at the same time, in a spatially defined and reproducible manner. This provides a framework for future studies that aim to address specific events and interactions occurring during human embryogenesis.

We envision that the integration of micropatterning technology with microfluidics could allow the generation of dynamic gradients of factors, controlled in space and time, giving the possibility to dissect the contribution of endogenous and exogenous factors in symmetry breaking within embryonic layers. In addition, it would be interesting to study how symmetry breaking would affect the cell sorting properties of different germ layers.

## Materials and Methods

### Micropatterning Technology

A 12-mm diameter glass coverslip was pre-treated with a plasma cleaner machine (Harrick Plasma) for 3 min (3 × 10^−1^ mbar) to oxidize the surface. Then 3–4 drops of solution A (10 μl of 3-(trimethoxysilyl) propyl methacrylate, 950 μl ethanol, and 50 μl acetic acid) were added onto the plasma-treated surface. After 3 min of incubation at room temperature, the coverslip was washed with ethanol two times and then let dry. A photo-patterning solution (solution B) was prepared right before use: 100 μl 20mg/100 μl IRGACURE 2959 (Ciba Specialty Chemicals) in methanol was diluted in 900 μl 8% (w/v) acrylamide (Sigma, 50 mM HEPES) and degassed for 20 min. Ten μl solution B was applied onto a 12-mm glass coverslip covered by a photo-mask and exposed for 40 s to UV irradiation with a distance of 5.5 cm between the glass coverslip and UV source. A functionalized glass coverslip was then washed with deionized water two times and stored at room temperature until use.

In the case of micropatterning within microfluidic devices, instead of using a 12-mm glass coverslip, a thick standard slide for microscopy was used and micropatterns were generated in correspondence with the areas that have been then covered by the microchannels.

Before cell seeding, the micropatterned glass coverslips were pre-coated with 50 μg/m Poly-L-lysin (Sigma) for 2 h at room temperature and 1% Matrigel overnight at 4°C. A washing procedure with MilliQ water and DPBS was performed after each coating. The glass coverslips were stored in the last wash solution at 4°C. After coating, the glass coverslips should be used within 2 weeks.

### Cell Culture

All experiments were performed with the H9 ESCs cell line. For routine culture maintenance, H9 ESCs were cultured in a StemMACSTM iPS-Brew XF pluripotent stem cell medium and passaged 1:5 to 1:10 every 3–5 days *via* EDTA dissociation. Culture plates were coated with 0.5% Matrigel (Corning) and incubated at room temperature for at least 2 h. Coated plates were stored at 4°C and pre-warmed at 37°C immediately before use.

### Micropatterned Ectoderm Induction

Human PSCs were pre-adapted to MEF-CM containing 20 ng/ml Fgf2 for one passage. Cells were detached as single cells and suspended in the MEF-CM medium supplemented with 10 μM Rock-inhibitor Y27632 (Miltenyi Biotech). The MEF-CM differentiation basal medium comprised DMEM high glucose (GIBCO), 20% KnockOut Serum Replacement (KSR, GIBCO), 1x GlutaMAX™ (GIBCO), 1x MEM NEAA (GIBCO), 100 μM β-mercaptoethanol (GIBCO), 2% B27-without vitamin A (GIBCO), and 1 mM pyruvate (Sigma) and conditioned overnight with inactivated MEFs. PSCs were seeded at 0.5 × 10 cells per well (Minn et al., 2020) onto the functionalized glass coverslips within 24-well plates. After 3 h, the micropatterns were washed twice with pre-warmed PBS to remove cells attached outside the micropatterns and the medium was replaced with an ectoderm induction medium containing a different combination of small molecules and was changed daily.

Three different basal media were used to induce ectoderm differentiation, which are as follows:

1) The DMEM differentiation basal medium containing DMEM/F12 (GIBCO), 15% KnockOut Serum Replacement (KSR, GIBCO), 1 × GlutaMAX™ (GIBCO), 1 × MEM NEAA (GIBCO), and 50 μM β-mercaptoethanol (GIBCO).

2) The MEF-CM differentiation basal medium.

3) N2B27 medium basal medium containing 25 ml DMEM/F12 medium, 25 ml neurobasal medium (GIBCO), 0.25 ml N2, 0.5 ml B27 without vitamin A, 0.5 ml GlutaMAX™, and 50 μM β-mercaptoethanol.

To induce ectoderm fate, the basal media were supplemented with different small molecules’ cocktail:

(1) 2 μM A83-01 (Tocris), 2 μM PNU-74654 (Tocris), and 2 μM dorsomorphin (Sigma) named APD.

(2) 10 μM SB431542 (Stemgent) and 100 nM LDN193189 (Sigma) named SB + LDN.

### Neural Ectoderm Induction in Conventional PSC Culture

H9 hESCs were seeded at high density as a single-cell onto Matrigel-coated Petri dishes. Upon attaining 90% confluence (usually the day after seeding), the medium was switched to MEF-CM supplemented with 1) 2 μM A83-01 (Tocris), 2 μM PNU-74654 (Tocris), and 2 μM dorsomorphin (Sigma) or 2) 10 μM SB431542 (Stemgent) and 100 nM LDN193189 (Sigma). Cells were fed daily and maintained till day 5 to evaluate the efficiency of neural induction.

### Meso-Endoderm Induction in Conventional PSC Culture

Meso-endoderm differentiation was induced in a 6-well Petri dish. H9 hESCs were seeded (1:5 passage) onto a 2.5% Matrigel-coated plate as clumps. When the cells reached 30–50% confluence (usually the day after split), the meso-endoderm induction was initiated by replacing the expansion medium with an induction medium comprising RPMI 1640 (GIBCO), 2% B27-ins (GIBCO), and 1 × MEM NEAA (GIBCO) supplemented with 100 ng/ml Activin A (R&D), 10 ng/ml BMP4 (R&D), 20 ng/ml bFGF (Peprotech), and 3 μM CHIR99021 (Miltenyi Biotech).

### Micropatterned Neuroectoderm Co-Culture With Meso-Endoderm

Micropatterned neuroectoderm cell fate was induced as described previously after 5 days of cell differentiation. Meso-endoderm cell induction was initiated on day 3 of neuroectoderm differentiation to keep in step. Meso-endoderm cells were cultured in a 6-well Petri dish with the RPMI medium, through single cell splitting, 0.25 × 10^6^ cells (in 0.5 ml MEF-CM medium) were seeded on top of the micropatterned neuroectoderm cells. During the ectoderm and meso-endoderm co-culture procedure, patterns were grown in the MEF-CM medium without any small molecules. The medium was changed daily for days.

### Immunocytochemistry and Image Acquisition

Cells were rinsed once with PBS, fixed in 4% paraformaldehyde for 30 min, and rinsed three times with PBS at room temperature. A blocking solution was made with 0.1% Triton-X and 5% normal donkey serum (Jackson ImmunoResearch, West Grove, PA) in PBS, and cells were incubated for 1 hour at room temperature before staining with primary antibodies in a blocking solution at 4°C overnight. The cells were then washed three times in PBS 0, 1% triton before being incubated for 2 h at room temperature with secondary antibodies, and 0.1 mg/ml DAPI (Invitrogen, Carlsbad, CA) in a blocking solution for 1 h. Finally, cells were washed three times in PBS 0, 1% triton and mounted using Vectashield Antifade Mounting Medium (Vector Laboratories, Burlingame, CA). All primary and secondary antibodies used are listed in Table 1.

**TABLE 1:** List of antibodies used.

Name	Brand and code (cat. no.)
AP-2a (3B5)	Santa Cruz Biotechnology, cat. no. sc-12726
Pax6	BioLegend, cat. no. 901301/PRB-278P
Sox17	R&D, cat. no. AF1924
Brachyury	R&D, cat. no. AF2085
Sox1	R&D, cat. no. AF3369
OCT4	Santa Cruz Biotechnology, cat. no. sc-5279
Sox2	EMD Millipore, cat. no. AB5603
Anti-p75	Promega, cat. no. G323A

Fluorescence images were acquired using a confocal TCS SP5 microscope (Leica Microsystems). Image processing was performed with ImageJ software (NIH).

### Quantification and Analyses

All micropatterning experiments and differentiation in standard culture conditions were performed at least three times. The data and analyses in each micropattern induction figure belong to one representative experiment. The sample size was not pre-determined and no statistical tests were used to determine the significance of results on micropatterned colonies. Circular colonies with a non-radially symmetric cell density pattern at the end of the treatment were excluded from analyses. The average intensity of a marker was calculated for each cell as the average of the immunofluorescence intensity in that cell normalized to the intensity of DAPI staining in the same cell.

## Data Availability

The raw data supporting the conclusion of this article will be made available by the authors, without undue reservation.
