# Supplementary material for: Human Pluripotent Stem Cell-Derived Micropatterned Ectoderm Allows Cell Sorting of Meso-Endoderm Lineages
Source: Front Bioeng Biotechnol. 2022 Jul 22;10:907159. doi: 10.3389/fbioe.2022.907159 (PMC9354750; doi:10.3389/fbioe.2022.907159)
Supplement: Supplementary file 1 [file DataSheet1.docx]

Supplementary Material

## Supplementary Figures


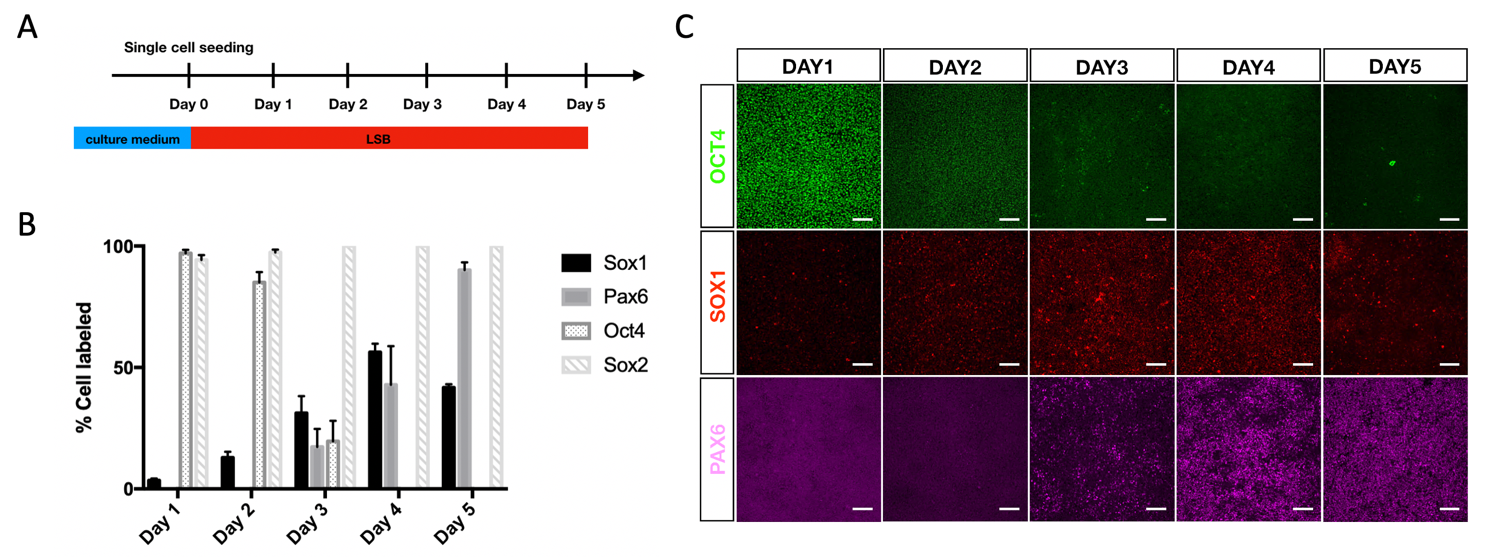


**Supplementary Figure 1.** **Dual SMAD inhibition on hPSCs. A)** Timeline and stimuli applied to 90% confluence monolayer culture of hPSCs. Culture medium= iPSC Brew medium. LSB = 100nM LDN193189 and 10uM SB = SB431542. **B)** Graph showing the percentage of cells expressing pluripotency marker Oct4, the neural ectoderm marker Sox1, Pax6, and the shared marker Sox2 during 5 days of neural induction. Error bar represents standard error. **C)** Immunostaining on hiPSCs for pluripotency marker OCT4 (green), and neural ectoderm-specific markers Sox1 (red) and Pax6 (purple) during the 5 days of neural induction. Scare bar = 100 μm. N=3 independent experiments.


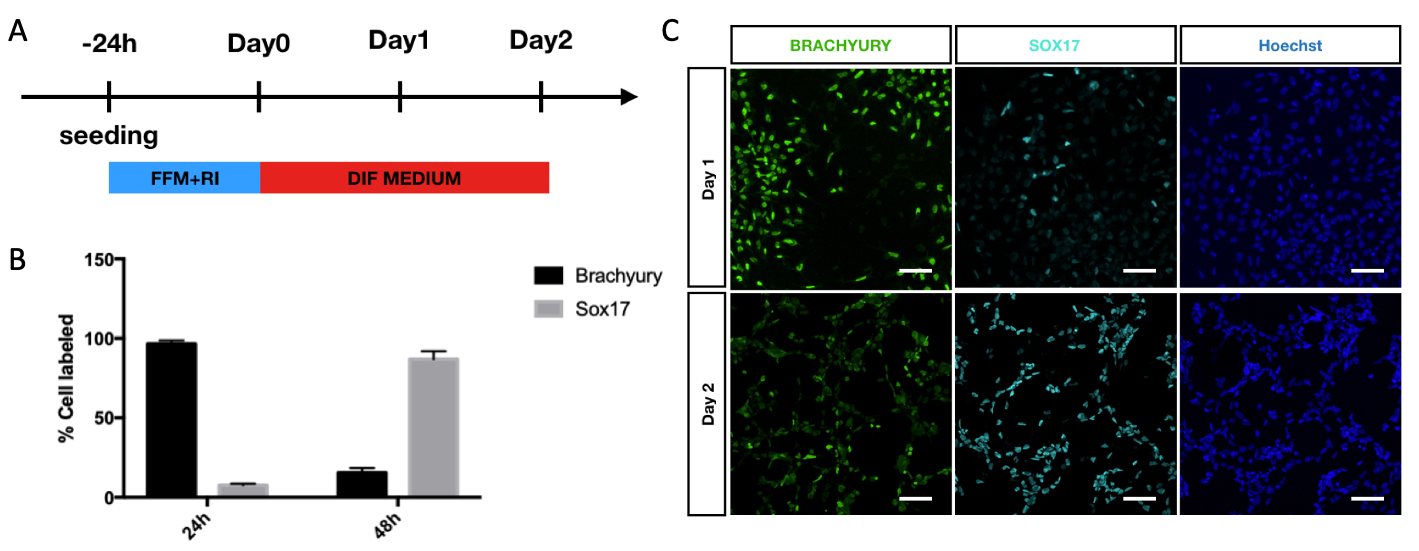


**Supplementary Figure 2. Meso-endoderm induction on hPSCs. A)** Timeline and stimuli applied to hPSCs. Culture medium= iPSC Brew medium. Diff medium=??. **B)** Graph showing the percentage of cells expressing mesoderm marker Brachiury/T and endoderm marker SOX17 at 24 and 48 hours of differentiation. Error bar represents standard error. **C)** Immunostaining on hPSCs for mesoderm marker Brachiury/T (green), and endoderm marker SOX17 (light blue) at 24 and 48 hours of differentiation. Nuclei are counterstained with blue. Scare bar = 100 μm. N= 3 independent experiments.
